# Phosphoproteomic profiling of lipopolysaccharide stimulated toll-like receptor pathways in macrophages

**DOI:** 10.1038/s41597-025-06108-z

**Published:** 2025-11-21

**Authors:** Doeun Kim, Sung Hwan Yoon, Nathan P. Manes, Aleksandra Nita-Lazar

**Affiliations:** Functional Cellular Networks Section, Laboratory of Immune System Biology, National Institute of Allergy and Infectious Diseases, National Institutes of Health, Bethesda, Maryland USA

**Keywords:** Toll-like receptors, Inflammation

## Abstract

Toll-like receptors (TLRs) are present on the surface of immune cells such as dendritic cells, macrophages, and natural killer cells. TLRs and other pattern recognition receptors (PRRs) are essential for the recognition of microbiological components. They trigger both innate and adaptive immune responses to defend against pathogenic microorganisms. Among the TLRs, TLR4 is one of the best-studied; it recognizes lipopolysaccharide (LPS) and initiates both TRIF- and MyD88-dependent signaling cascades. Regulators of TLR4 signaling, including numerous protein kinases, have a key role in innate and adaptive immune responses. Although TLR4 signaling pathways have been investigated using phosphoproteomics by mass spectrometry using data-dependent acquisition (DDA), the phosphoprotein landscape of the TLR4 signaling remains poorly incomplete, partly due to the technical limitations of DDA. To address this, we utilized data-independent acquisition (DIA) mass spectrometry to deeply explore phosphorylation dynamics within the LPS-stimulated TLR4 signaling pathway in macrophages.

## Background & Summary

The innate immune system acts as an initial defense mechanism against pathogens, initiating and regulating the inflammatory response^1,2^. Pathological and potentially lethal disorders like sepsis and chronic inflammatory diseases can arise from an excessive response to dysregulated inflammation^3–6^. Macrophages are myeloid-derived innate immune cells that are essential for controlling the inflammatory response. Pathogen-associated molecular patterns (PAMPs), which include bacterial, viral, and fungal components, are recognized by innate immunological pattern recognition receptors (PRRs)^7,8^. When PRRs are activated, signal transduction is initiated, leading to the expression of pro-inflammatory genes. Toll-like receptors (TLRs) belong to the family of PRRs recognizing PAMPs located on the cell surface or inside endosomes. TLR4 is a well-studied TLR that detects lipopolysaccharide (LPS), a key component of the cell wall of gram-negative bacteria. The two TLR4 signal transduction pathways are the MyD88-dependent and the TRIF-dependent signaling cascades. TRIF-dependent signaling primarily generates type-I interferons, whereas MyD88-dependent signaling results in the production of pro-inflammatory cytokines and chemokines^9–12^. The TLR4 signaling cascades involve in numerous molecular processes, including complex formation with activating and inhibitory proteins, post-translational modifications (PTMs) such as phosphorylation and ubiquitination, and protein relocalization^13–15^. Protein phosphorylation on serine, threonine, and tyrosine (S/T/Y) is a critical regulatory PTM. Due to its rapid dynamics, S/T/Y phosphorylation functions as a molecular switch for signal transduction, enabling cells to connect extracellular signals to numerous physiological processes, including the activation of the innate and adaptive immune systems in response to pathogens, which has been previously examined at the phosphoproteome level^16–19^. In 2014, we investigated phosphoprotein dynamics during signaling cascades activated by TLR2, TLR4, and TLR7 ligands, utilizing high-resolution mass spectrometry with data-dependent acquisition (DDA)^20^. This investigation was notable due to the lack of phosphoproteomic studies focused on TLRs other than TLR4, and the absence of comparative analyses of global phosphoprotein signatures across different TLRs. However, it is still uncertain what molecular processes underlie alterations in phosphoproteome dynamics that result in cytokine storms.

Compared to the traditional DDA, the latest developments in data independent acquisition (DIA) mass spectrometry offer improved sensitivity and protein coverage^21,22^. As shown in a multi-laboratory evaluation study, DIA allows the quantification of proteins with low variation and excellent reproducibility^23–25^. In theory, DIA analyzes all peptides without bias including more than 90% of all tryptic peptides within the chosen m/z windows, which are usually between 400 and 1,200 m/z^26,27^. The capabilities of DIA are continually increasing and approaching proteome-wide levels. Furthermore, as demonstrated by recent studies^28–31^, DIA can be used to quantify PTMs in a high throughput manner. Although DIA-based proteomics has been employed by several research groups to elucidate protein dynamics and phosphorylation events in the LPS signaling pathway, comprehensive insights remain limited^32,33^. To broaden our understanding of phosphorylation during TLR4 signaling in macrophages, we utilized and compared the results of DDA-MS and DIA-MS.

## Methods

### Cell culture

Immortalized bone marrow-derived macrophages (iBMDMs) were kindly provided by Dr. Eicke Latz^34^. iBMDMs were cultured in DMEM (Gibco, USA) supplemented with 10% FBS (Gibco), in a 5% CO_2_ incubator at 37 °C, maintained at low densities and passaged until reaching the confluent state, usually every 3–4 days on sterile tissue culture plates. All the cell cultures were done at 6–10 passages. All sample groups have five biological replicates. iBMDMs were stimulated with 100 ng/mL of LPS (LPS from *Escherichia coli* O127:B8, Sigma, USA), as previously described^35–37^, then washed with ice-cold 1x PBS (Quality biological Inc., USA) three times.

### Sample preparation for LC-MS

Phosphoproteomic and global proteomic samples were prepared according to Di, Yi, *et al*.^38^ and our previous publications^20,39^. Briefly, ice-cold 1x PBS washed iBMDMs were lysed in 100 mM ammonium bicarbonate pH 8, 10 M urea, halt protease inhibitor and phosphatase inhibitor cocktails (Thermo Fisher Scientific, USA) using bioruptor (Diagenode, Seraing, Belgium). Supernatants were collected after centrifugation at 20,000 g for 1 hr at 7 °C. Total protein concentration was determined by BCA protein assay (Thermo Fisher Scientific). For each sample, 2 mg of protein was reduced by adding DTT (Millipore Sigma, Merck KGaA, Darmstadt, Germany) at 56 °C for 1 hr and final concentration was 10 mM. Then, alkylation was done by adding IAM (Millipore Sigma, USA) at RT in darkness for 45 min and final concentration was 20 mM. Sample solutions were diluted to 1 M urea by adding 100 mM ammonium bicarbonate pH 8 (Thermo Fisher Scientific). Sequencing grade modified trypsin (Promega, USA) was added to each sample (1:25 w:w enzyme:protein) and samples were incubated in thermomixer at 37 °C overnight with gentle shaking. Trypsin digestion was terminated by adding formic acid to pH < 3 followed by desalting using a Sep-Pak C18 cartridge following the manufacturer’s protocol (Waters, USA).

To conduct global proteomic analyses, 5% of each eluate was dried in a SpeedVac vacuum concentrator (Thermo Fisher, USA). The remaining 95% of each eluate underwent phospho-enrichment using TiO_2_ and Fe-NTA kits sequentially (Pierce High Select, Thermo Fisher Scientific) following the manufacture’s protocols. The two eluents from each phospho-enrichment column were pooled and dried in a lyophilizer (SP scientific, USA) overnight. Dried phosphopeptides were loaded onto SDB (GL sciences) and graphite desalting spin columns (Thermo Fisher Scientific) sequentially to purify phosphopeptides, then dried using a SpeedVac vacuum concentrator (Thermo Fisher Scientific) without heating. All peptide samples were resuspended in the solution (LC-MS grade water with 0.1% v/v formic acid, 2% v/v ACN) and the peptide concentration was measured using a NanoDrop (DeNovix Inc., USA).

### Liquid chromatography tandem mass spectrometry (LC-MS/MS)

An Orbitrap Eclipse Tribrid mass spectrometer (Thermo Fisher Scientific) coupled to an UltiMate 3000 LC system (Thermo Fisher Scientific) was used for LC-MS/MS experiments. One μg of total peptides was injected for each LC-MS/MS analysis. LC-MS technical duplicates were performed for each sample. Peptides were trapped on an Acclaim C18 PepMap 100 trap column (5 µm particles, 100 Å pores, 300 µm i.d. x 5 mm, Thermo Fisher Scientific) and separated on a PepMap RSLC C18 column (2 µm particles, 100 Å pores, 75 µm i.d. x 50 cm, Thermo Fisher Scientific) at 40 °C. The LC steps were: 98% mobile phase A (0.1% v/v formic acid in H_2_O) and 2% mobile phase B (0.1% v/v formic acid in ACN) from 0 to 5 min, 2% to 35% linear gradient of mobile phase B from 5 to 155 min, 35% to 85% linear gradient of mobile phase B from 155 to 157 min, 85% mobile phase B from 157 to 170 min, 85% to 2% linear gradient of mobile phase B from 170 to at 172 min, 2% of mobile phase B from 172 to 190 min. The LC flow rate was set to 225 nL/min. Eluted peptides were electrospray ionized in positive ion polarity at 2.1 kV.

For DDA, MS^1^ full scans were recorded in the range of m/z 375 to 1,500 with a resolution of 120,000 at 200 m/z using the Orbitrap mass analyzer. Automatic gain control and maximum injection time were set to standard and auto, respectively. Top 3 sec data dependent acquisition DDA mode was used to maximize the number of MS^2^ spectra from each duty cycle. Higher-energy collision-induced dissociation (HCD) was used to fragment selected precursor ions with normalized collision energy of 27. MS^2^ scans were recorded using an automatic scan range with a resolution of 15,000 at 200 m/z using the Orbitrap mass analyzer. After selection, precursors were excluded from selection for 60 sec. Automatic gain control and maximum injection time were set to standard and auto, respectively.

The DIA-MS method consisted of one MS^1^ full scan and 40 MS^2^ scans. MS^1^ full scans were recorded in the range of m/z 380 to 860 with a resolution of 120,000 at 200 m/z using the Orbitrap mass analyzer. The MS^2^ scans had isolation window of 13 m/z and window overlap of 1 m/z. The MS^2^ scan range was set to m/z of 150 to 2,000. HCD was used to fragment ions with normalized collision energy of 27. Automatic gain control and maximum injection time were set to standard and 54 ms, respectively.

### LC-MS/MS data analysis

For the DDA results, peptide identification and label-free quantification was performed using Proteome Discoverer v. 3.1 (Thermo Fisher Scientific) using the Chimerys search engine. Spectronaut v.19.0 (Biognosis, Zurich, Switzerland) was used for the DIA data. The raw files were searched against the mouse reference proteome database downloaded from UniProt^40^ (https://www.uniprot.org/proteomes/UP000000589, download date 2024.3.15). For the global proteomics analyses, the peptide spectra matches (PSMs) and protein identification false discovery rate (FDR) was set to 1%. Maximum number of missed cleavages allowed up to two. Further, proteins were discarded unless they were identified by two or more unique peptides and four or more PSMs. For the DDA data, match-between-runs was used, and for the DIA data, the feature identification FDR was 1%. Each protein abundance value was the sum of the corresponding peptide abundance values. For the phosphoproteomic analyses, the PSM and phosphorylation site FDR was set to 1%, the phosphorylation localization probability was set to >0.75. In addition, four or more PSMs were required for each phosphopeptide.

Both the DDA and DIA phosphopeptide abundance values were log_2_ transformed, averaged across the two technical duplicates, and partitioned into two data matrixes using Perseus 1.6.15.0^41^. Each “primary” data matrix consisted of the phosphorylation sites having at least 40% valid values (i.e., < 60% missing values) in all of the experimental groups, whereas each “secondary” data matrix consisted of the remaining abundance data. Each primary data matrix underwent imputation using NAguideR^42^ after intra-level normalization by median subtraction. Among the numerous imputation methods available within NAguideR, we assessed ten: zero, minimum, perseus imputation, k-nearest neighbor (knn), sequential knn (seqknn), quantile regression (qr), local least squares (lls), glmnet ridge regression (grr), multiple imputation bayesian linear regression (mice-norm), and random forest (rf). We selected seqknn based on rank under four classic criteria (Figure S1 and S2). Each secondary data matrix was averaged by arithmetic mean across the biological replicates, with filtering such that the minimum valid values for all experimental groups was two and removed rows if all groups have no values. Principal component analyses (PCAs) of both data matrixes were generated by the R package (*scatterplot3d*). Statistical analyses were conducted by one-way ANOVA and Tukey’s honest significant difference test by R package (*tidyverse*). The Gene Ontology (GO) and Kyoto Encyclopedia of Genes and Genomes (KEGG) pathway enrichment analyses of significantly regulated phosphoproteins were conducted with R package (*clusterProfiler*)^43–46^.

### SDS-PAGE and Western blot

Total protein concentration was determined using BCA protein assay (Thermo Fisher Scientific). Thirty μg of each sample for immunoblot assay and 10 μg of each sample for Coomassie blue staining were loaded into Bolt 4 to 12% Bis–Tris polyacrylamide gels (Invitrogen, USA) and run at 200 V for 45 min. The proteins were transferred to the PVDF membrane (Thermo Fisher Scientific) at 30 V for 75 min. Briefly, the membranes were blocked with 5% BSA for three hours, incubated with specific primary antibodies (phospho-JNK (Cell signaling technology (CST, USA) #4668S), phospho-p38 (CST #9211S), phospho-p65 (CST #3033S), β-actin (CST #4970S), pan-phospho-serine (Abcam, Cambridge, UK; #ab9332), and pan-phospho-threonine/tyrosine (CST #9381)) overnight followed by incubation with secondary antibodies for 1 hr at RT. The blots were developed using Clarity Max Western ECL substrate (Bio-Rad, USA). The results were visualized using a ChemiDoc imaging system (Bio-Rad).

## Data Records

All phosphoproteomics and global proteomics LC-MS data have been deposited at the ProteomeXchange Consortium^47^ via the PRIDE partner repository^48^ with the dataset identifier PXD064064^49^. This dataset consists of raw LC-MS files and processed files including the Proteome Discoverer v. 3.1 output file, and Spectronaut v. 19.0 output file.

## Technical Validation

**Fig. 1:**
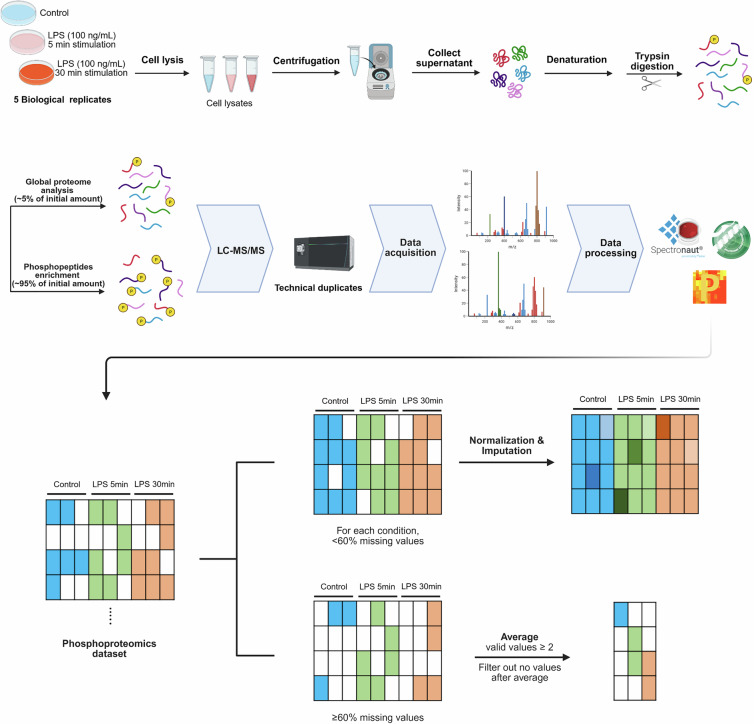
Overview of the phosphoproteomic and global proteomic workflows. Time-course LPS-stimulated iBMDMs were prepared (five biological replicates) for phosphoproteomic and global proteomic analyses. LC-MS/MS technical duplicates were performed, followed by the generation of phosphoproteomic and global proteomic datasets.

**Fig. 2:**
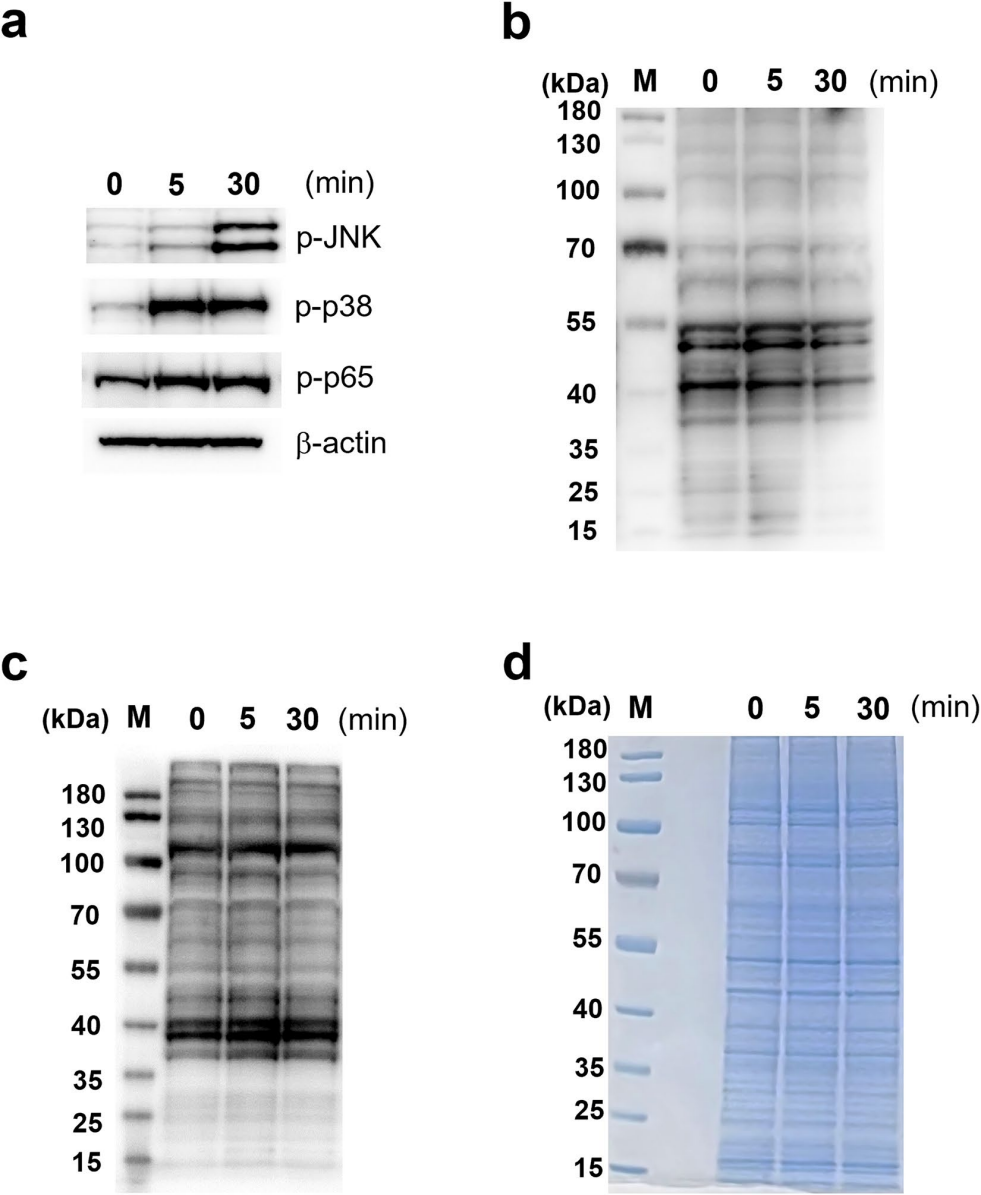
Phosphorylation in LPS-stimulated iBMDMs over a time-course. (**a**) Western blot assays of phospho-JNK, phospho-p38, and phospho-p65. β-actin was used as a loading control. (**b**) Western blot assay using a pan-phosphoserine primary antibody. (**c**) Western blot assay using a pan-phosphothreonine and phosphotyrosine primary antibody. (**d**) Coomassie blue staining as a loading control. M denotes the protein molecular weight markers.

To deeply profile phosphorylation dynamics during LPS stimulation, LC-MS based phosphoproteomics was used to study LPS-stimulated iBMDMs (Fig. 1). We stimulated iBMDMs with 100 ng/mL of LPS up to 30 min to investigate the signaling cascades that result in production of inflammatory cytokines. Western blots were performed against phosphorylated forms of three well-studied protein kinases in TLR4 signaling pathways: p38, JNK, and p65 (Fig. 2a). These proteins were phosphorylated in response to LPS stimulation, as expected. Additionally, pan-phospho-serine and pan-phospho-threonine/tyrosine antibodies, which capture all phospho-serine/threonine/tyrosine residues, were used to measure the global protein phosphorylation level across the LPS stimulation time-course (Figs. 2b and 2c). We used the same samples for Coomassie blue staining to ensure that the gel loading masses were the same for phospho-serine/threonine/tyrosine detection (Fig. 2d). Globally, protein phosphorylation levels were similar at all time points.

To demonstrate that the measured protein phosphorylation dynamics were not influenced by global proteomic changes, we conducted global proteomic analyses and measured the abundance changes across all sample groups using both the DDA and DIA methods. From our global proteomics analyses, across all experimental groups analyzed using DDA we identified 7,547 proteins and 100,650 peptides, while the DIA analyses yielded 7,442 proteins and 101,433 peptides. We calculated and graphed each sample’s pairwise correlation for the DDA and DIA datasets to ensure the precision of our global proteomic results. The correlation among samples of the global proteomic datasets demonstrated a high correlation coefficient ( > 0.969 in DDA, > 0.987 in DIA), indicating the high quality of the global proteomics dataset (Figure S3 and S4). Also, we found that only a small proportion of proteins showed significant changes in abundance (Figure S5 and S6). Although time-course LPS stimulation led to differentially expressed proteins in the global proteome, there was only up to 0.5% overlap with the phosphoproteome. These findings indicate that changes in phosphorylation abundance occur independently of changes in total protein abundance.

**Fig. 3:**
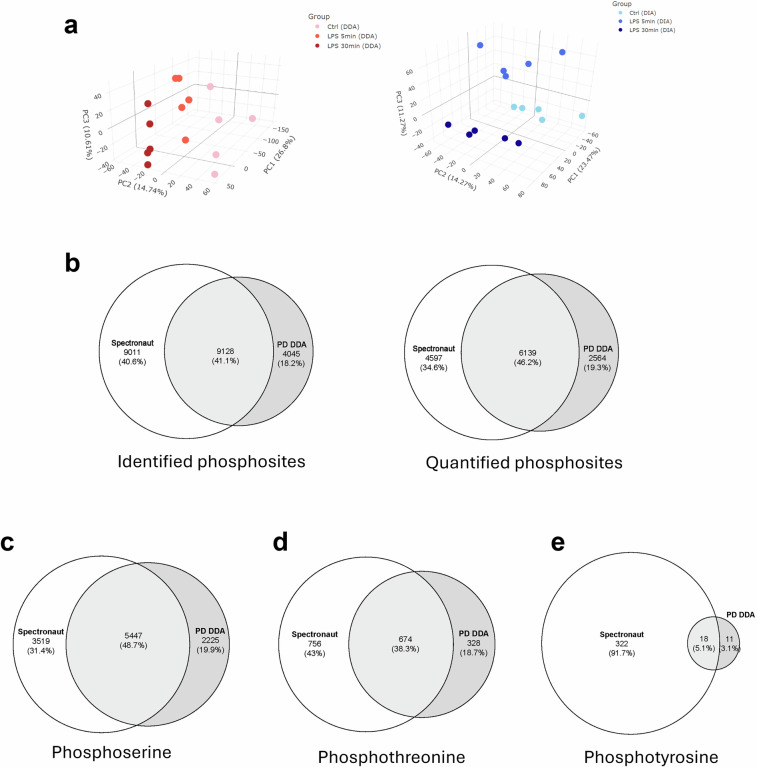
Comparison of the number of identified and quantified phosphosites between the DDA and DIA datasets across all of the experimental groups. Here, the primary data matrices were analyzed. (**a**) PCA plot of DDA and DIA quantified phosphopeptide datasets. (**b**) Venn diagrams of identified and quantified (right) phosphosites between the Proteome Discoverer (PD) DDA and Spectronaut DIA datasets. (**c**) Venn diagram of the quantified serine phosphorylation sites. (**d**) Venn diagram of the quantified threonine phosphorylation sites. (**e**) Venn diagram of the quantified tyrosine phosphorylation sites.

Label-free quantification was used to measure changes in protein phosphorylation during LPS stimulation at three time points (0, 5, and 30 min). PCA plots show that the LPS stimulation time points were clearly separated, demonstrating that there were significant differences between the experimental groups in the phosphoproteomic dataset (Fig. 3a). We identified 13,894 phosphopeptides using DDA and 21,761 phosphopeptides using DIA. After partitioning the DDA and DIA datasets into primary and secondary data matrices, intra-level normalization by median subtraction, and missing value imputation for primary data matrices, there were 10,023 and 12,710 quantitated phosphopeptides in the DDA and DIA results, respectively. To assess the precision of the phosphoproteomics quantitation, pairwise correlation coefficients were calculated, both before and after seqKNN imputation (Figure S7 to S10). The mean DDA and DIA correlation coefficients were 0.925 and 0.955, respectively, indicating that our phosphopeptide quantitation was highly precise.

Within the DDA and DIA datasets, 13,173 and 18,139 phosphosites were identified after filtration, respectively. A total of 9,128 overlapping phosphosites were identified. From the quantitated phosphopeptides, the DDA and DIA datasets contained 8,703 and 10,736 quantitated phosphosites, respectively (Fig. 3b). Venn diagrams indicated that 41% and 46% of phosphosites overlapped within the sets of identified and quantified phosphosites (respectively) between the DDA and DIA datasets (Fig. 3b). Approximately 19% of phosphosites were identified exclusively through DDA, while roughly 38% were detected exclusively by DIA. Among the quantified phosphosites, serine was the predominant phosphorylated amino acid (Fig. 3c), as has been demonstrated by numerous previous studies^50–53^. Serine phosphorylation was detected at 7,672 and 8,966 phosphosites by DDA and DIA, respectively. The analysis revealed that 88.2% of total quantified phosphosites by DDA were serine phosphorylation, while it was 83.5% for DIA. Quantified threonine phosphorylation was discovered on 1,002 sites in the DDA dataset and 1,430 sites in the DIA dataset (Fig. 3d). Quantified threonine phosphorylation sites accounted for 11.5% and 13.3% of the total as detected by DDA and DIA, respectively. The quantity of phosphorylated tyrosine exhibited a notable difference between the DDA and DIA datasets. In the DDA dataset, phosphotyrosine was detected at 29 sites, representing 0.3% of the quantified phosphosites in DDA. The DIA dataset contains 340 phosphotyrosine sites within quantified phosphopeptides, representing 3.2% of the total phosphorylation sites, which is comparable to the estimated phosphotyrosine site percentage. Compared to the DDA approach, the DIA method enabled nearly 10-fold increase in the phosphotyrosine quantitation without typical phosphotyrosine-specific antibody-based enrichment.

**Fig. 4:**
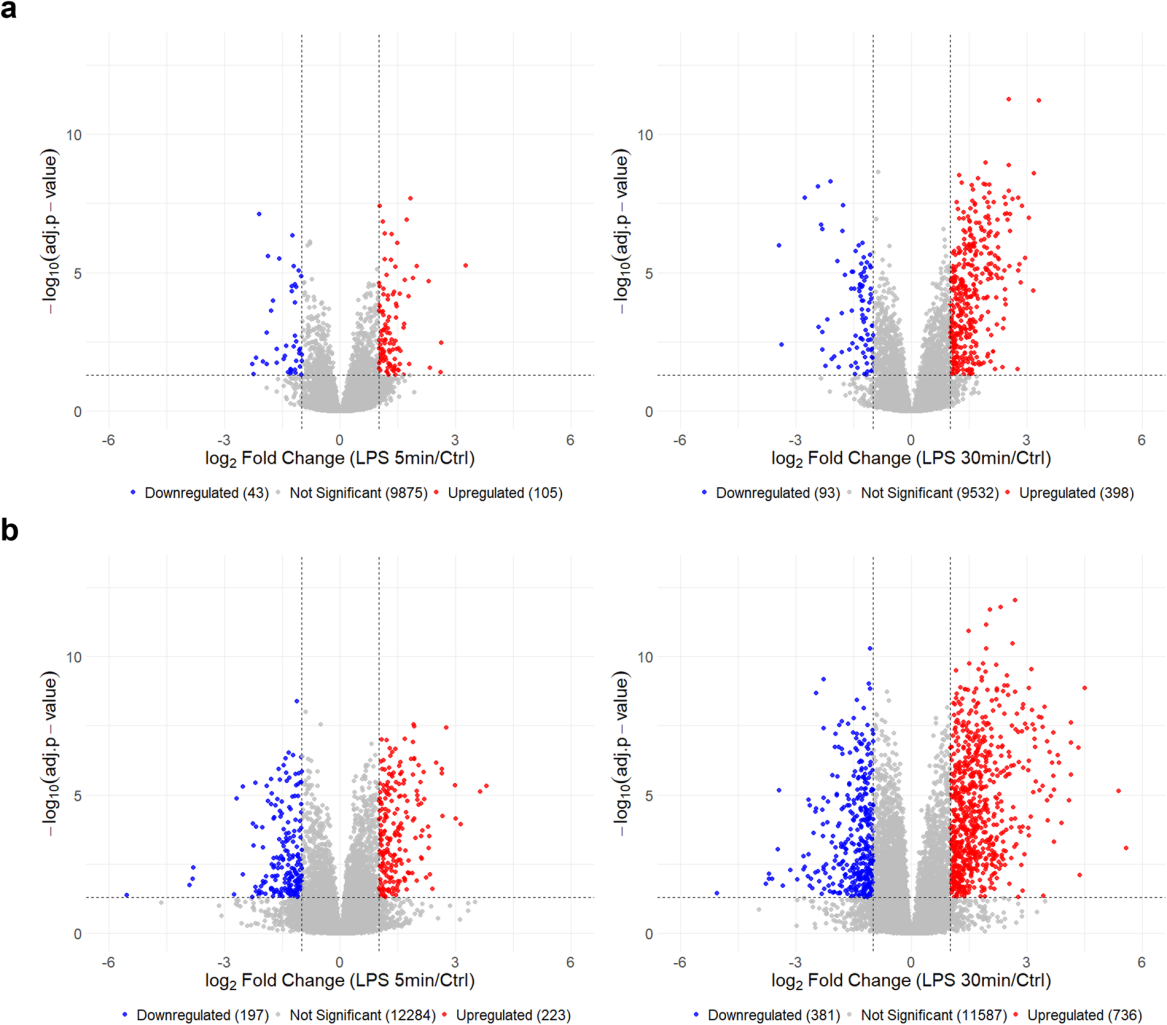
Volcano plots of quantified phosphopeptides obtained using DDA and DIA. Here, the primary data matrices were analyzed. Statistical analyses were conducted by one-way ANOVA and Tukey’s honest significant difference test **(a)** Volcano plots of the quantified phosphopeptides from the DDA dataset. The left panel depicts the 5 min LPS vs. Ctrl results, and the right panel depicts the 30 min LPS vs. Ctrl results. **(b)** Volcano plots of the quantified phosphopeptides from the DIA dataset. The left panel depicts the 5 min LPS vs. Ctrl results, and the right panel depicts the 30 min LPS vs. Ctrl results. Blue dots indicate downregulated phosphopeptides, and red dots represent upregulated phosphopeptides. The cut-off was set to ±1 for the log₂ fold changes, and the adjusted p < 0.05.

Volcano plots were generated to visualize the differential expression of the quantified phosphopeptides between the LPS-stimulated and control groups. Phosphopeptides with a log₂ fold change greater than 1 or less than -1 and an adjusted p-value less than 0.05 were considered significantly upregulated or downregulated, respectively. In the DDA dataset, 105 phosphopeptides were significantly upregulated and 43 were significantly downregulated in the 5 min LPS stimulation condition (Fig. 4a). In the 30 min LPS stimulation group, 398 phosphopeptides were significantly upregulated and 93 were significantly downregulated. The DIA dataset contained 223 significantly upregulated and 197 significantly downregulated phosphopeptides in the 5 min LPS stimulation group (Fig. 4b). Further, 736 phosphopeptides were significantly upregulated and 381 were significantly downregulated following 30 min LPS stimulation. Interestingly, DIA quantified over 2-fold more significantly regulated phosphopeptides compared to DDA in all of the LPS-stimulation time points.

**Fig. 5:**
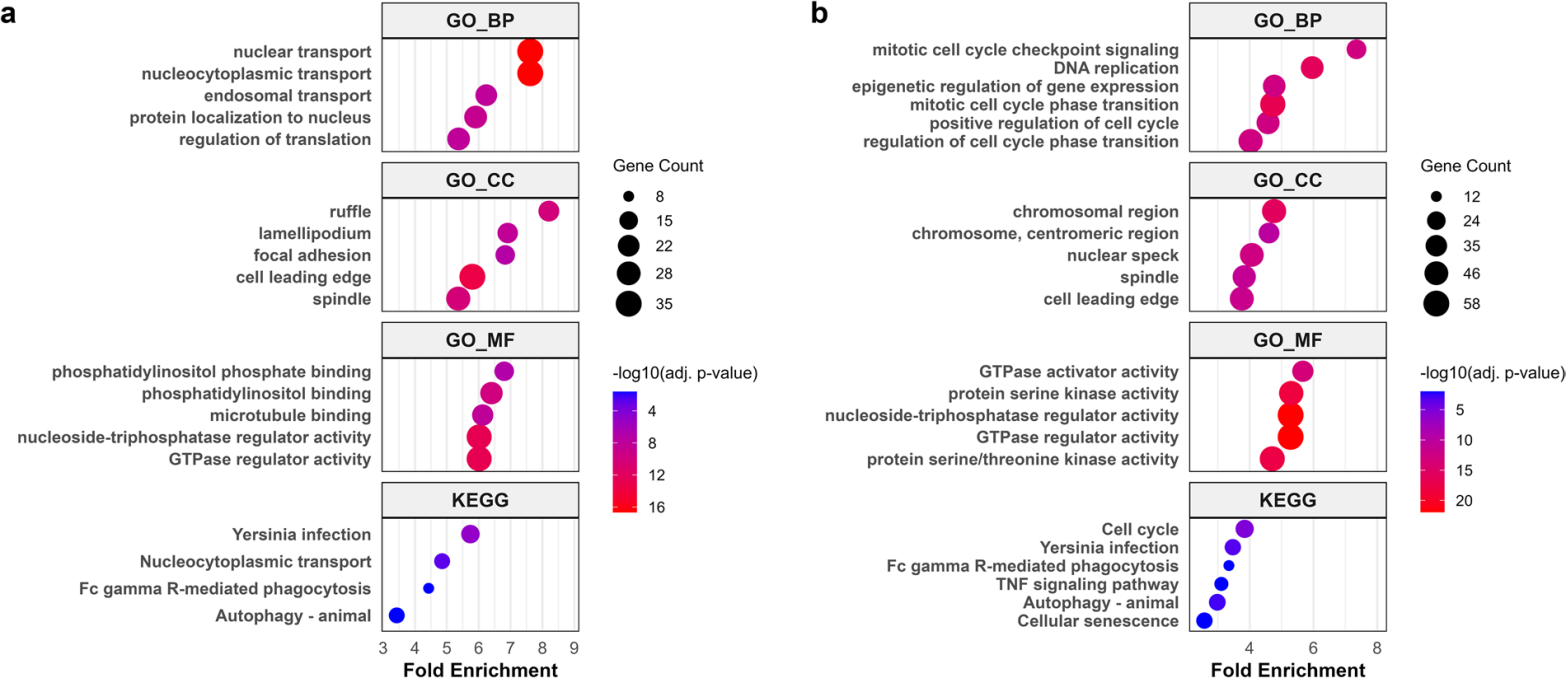
GO and KEGG enrichment analyses results of significantly regulated phosphoproteins in 30 min LPS stimulation group. Here, the primary data matrices were analyzed. (**a**) Dot plots of the Gene Ontology (GO) and (KEGG) enrichment results from significantly of the regulated phosphoproteins in the DDA dataset. (**b**) Dot plots of the GO and KEGG enrichment results from significantly regulated phosphoproteins in the DIA dataset. GO biological process (GO_BP), GO cellular components (GO_CC), GO molecular functions (GO_MF), and KEGG enrichment analyses were conducted using clusterProfiler.

To perform the functional enrichment analyses for significantly regulated phosphoproteins in time-course LPS stimulation, we utilized using R package called clusterProfiler. Using clusterProfiler, we did GO and KEGG enrichment analyses and the GO analysis was applied to three ontologies: biological process (BP), cellular component (CC), and molecular function (MF). GO and KEGG terms with an adjusted p-value below 0.05 were considered significantly enriched. The top 5 terms in each category are illustrated using dot plots. When we checked gene count in GO and KEGG terms of DDA and DIA dataset, most of terms in DIA have over 2-fold more gene count compared to DDA across all LPS stimulation time points. In the 30 min LPS stimulation group, top 5 enriched GO terms are involved in regulation of cell cycle and kinase/phosphatase binding and activity in both DDA and DIA datasets (Fig. 5). In the top 5 enriched KEGG terms, inflammatory-related terms such as Yersinia infection, Fc gamma R-mediated phagocytosis, and Autophagy were enriched in both datasets as we expected. In here, DIA gives one more KEGG term named TNF signaling pathway within top 5 terms. Along with 30 min LPS stimulation group, GO enrichment analysis in 5 min LPS stimulation group showed the similar terms related to regulation of cell cycle (Figure S11). The GO and KEGG enrichment analyses indicate that significantly regulated phosphoproteins after LPS stimulation are highly involved in innate immune response.

**Fig. 6:**
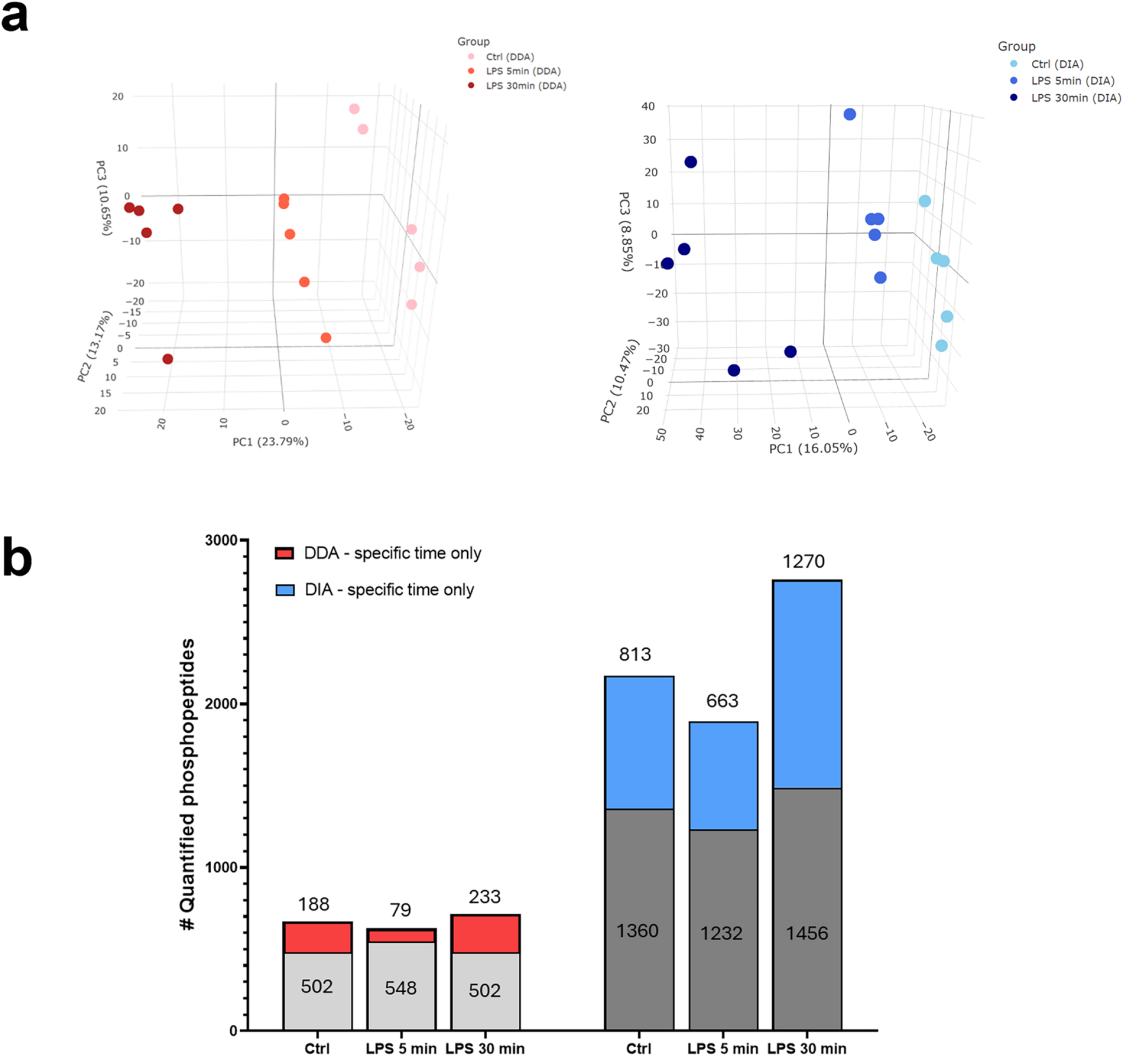
Comparison of the number of phosphopeptides quantified from specific time-point LPS stimulation groups in DDA and DIA datasets. Here, the secondary data matrices were analyzed. (**a**) PCA plot of the DDA and DIA quantified phosphopeptide datasets. (**b**) Stacked bar charts of the number of DDA and DIA quantified phosphopeptides. The number on top of each bar denotes the number of time-specific quantified phosphopeptides, and these are depicted by red and blue bar regions. The gray bar regions depict phosphopeptides identified in two experimental conditions, and absent from one experimental condition.

The secondary matrices, which were of the time-point-specific phosphopeptides, demonstrated clear separation between each experimental group in PCA plots (Fig. 6a). A total of 1,257 quantified phosphopeptides were identified in the DDA dataset, while the DIA dataset contained 4,867 quantified phosphopeptides. Among the LPS stimulation time points, the 30 min LPS stimulation group exhibited the highest number of quantitated phosphopeptides (Fig. 6b). The 5 min LPS stimulation group exhibited the lowest number of quantified phosphopeptides in both datasets. The number of quantitated phosphopeptides exclusively in the control group was 187 in the DDA dataset and 813 in the DIA dataset. The number of quantitated phosphopeptides exclusively in the 5 min LPS stimulation group was 79 in the DDA dataset and 663 in the DIA dataset. The number of quantitated phosphopeptides exclusively in the 30 min LPS stimulation group was 233 in the DDA dataset and 1,270 in the DIA dataset. Across all LPS stimulation time points, DIA quantified at least four-fold the phosphopeptides compared to DDA within the secondary data matrices.

In conclusion, this study provides a comprehensive and technically robust analysis of phosphorylation dynamics in LPS-stimulated iBMDMs using both immunoblotting and high-resolution mass spectrometry techniques. Time-course stimulation with 100 ng/mL LPS revealed differential phosphorylation of key TLR4 signaling kinases (p38, JNK, p65), while total phosphorylation levels assessed via pan-phospho antibodies were relatively constant. To ensure phosphoproteomic data accuracy, a loading control was used for immunoblotting (total β-actin). Likewise, global proteomics was incorporated into our DDA and DIA experimental methods to account for the total abundance of each protein. DDA and DIA approaches for phosphoproteomic analyses using extensive data filtration ensured that the observed phosphorylation changes were biologically relevant and not artifacts, establishing a strong technical approach for measuring LPS-stimulated phosphorylation dynamics. Although the DDA and DIA datasets are complementary, DIA detected 2-fold significantly up/down-regulated phosphopeptides compared with DDA. Surprisingly, the number of quantified phosphotyrosine peptides was more than 10-fold higher in the DIA dataset compared to DDA. In contrast to serine and threonine phosphorylation, tyrosine phosphorylation is much less abundant and more transient, making it challenging to detect in phosphoproteomic analyses. In the DIA dataset, several tyrosine-phosphorylated proteins were exclusively identified, including not only well-established regulators of the TLR4 signaling pathway (Tab2, Mapk3, and p38), but also RNA splicing factors (Ddx39b, Rbm17, Srsf1, Srsf6, Slu7, Tra2a, Tra2b, Ncbp2) and regulators of the ErbB signaling pathway (Crk, Grb2, Nck2, Pak4), which have been extensively studied in the context of cancer. These findings highlight the enhanced sensitivity and precision of DIA-based phosphoproteomics, particularly for capturing low-abundance and transient modifications such as phosphotyrosine. In a follow-up study, we will investigate the temporal dynamics of phosphoproteins using a DIA approach and validate identified tyrosine-phosphorylated proteins through biological assays.

## Supplementary information

Supplemental figures

## Data Availability

No custom code was used in this work. The software and R packages required for data processing have been described in detail in the methods section (LC-MS/MS data analysis).
