# Supplementary material for: Phosphoproteomic profiling of lipopolysaccharide stimulated toll-like receptor pathways in macrophages
Source: Sci Data. 2025 Nov 21;12:1856. doi: 10.1038/s41597-025-06108-z (PMC12638788; doi:10.1038/s41597-025-06108-z)
Supplement: Supplementary file 1 — Supplemental figures [file 41597_2025_6108_MOESM1_ESM.docx]

**Supplemental figures**


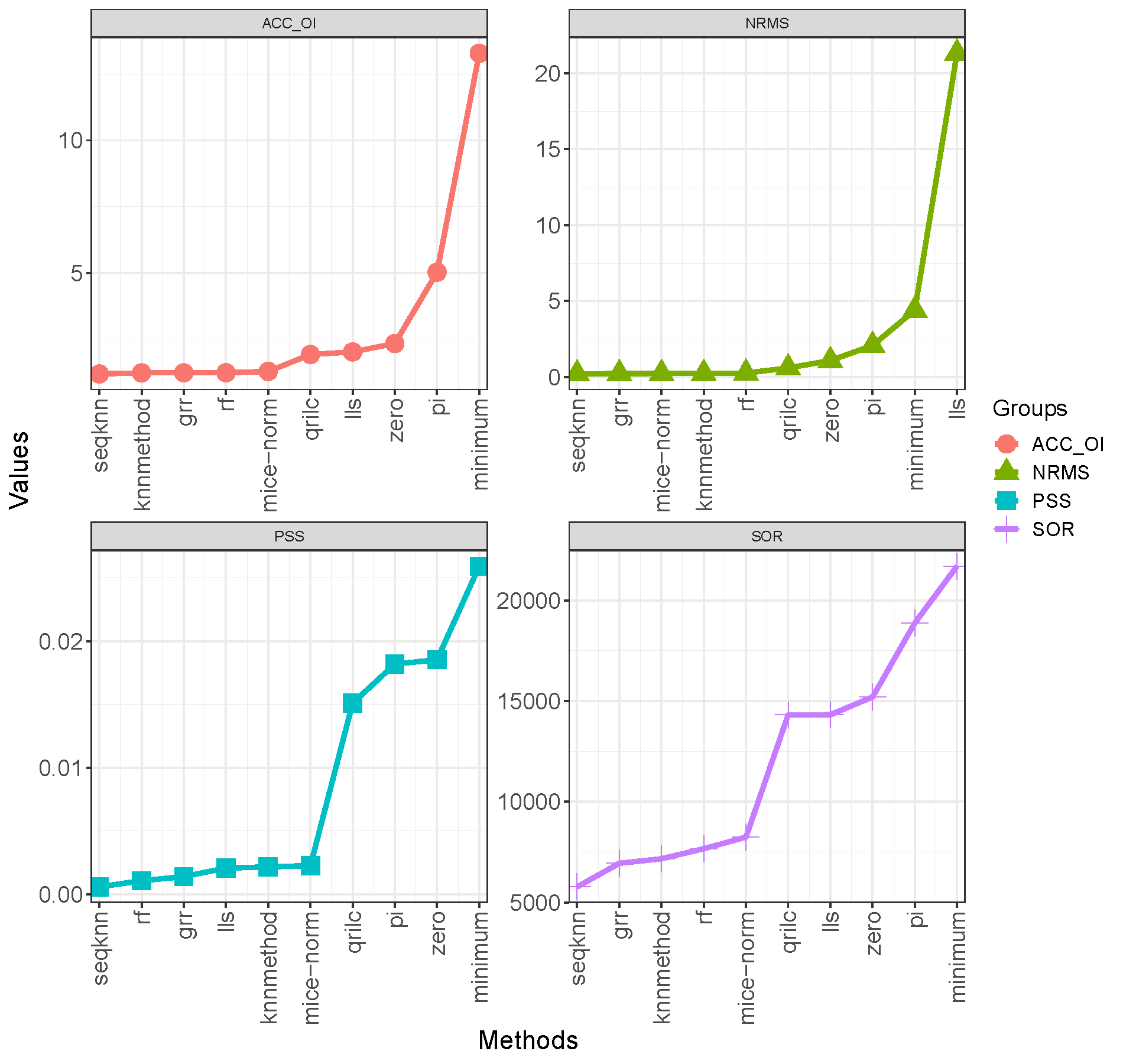


**Figure S1. Benchmarking of the imputation methods for the primary matrix for the DDA dataset.** Ten imputation methods were assessed: zero, minimum, perseus imputation, K-nearest neighbor (knn), sequential knn (seqknn), quantile regression (qr), local least squares (lls), glmnet ridge regression (grr), multiple imputation bayesian linear regression (mice-norm), and random forest (rf). For each graph, the methods are sorted by value. seqknn was assessed to be the top ranked method and was used for the downstream analyses.


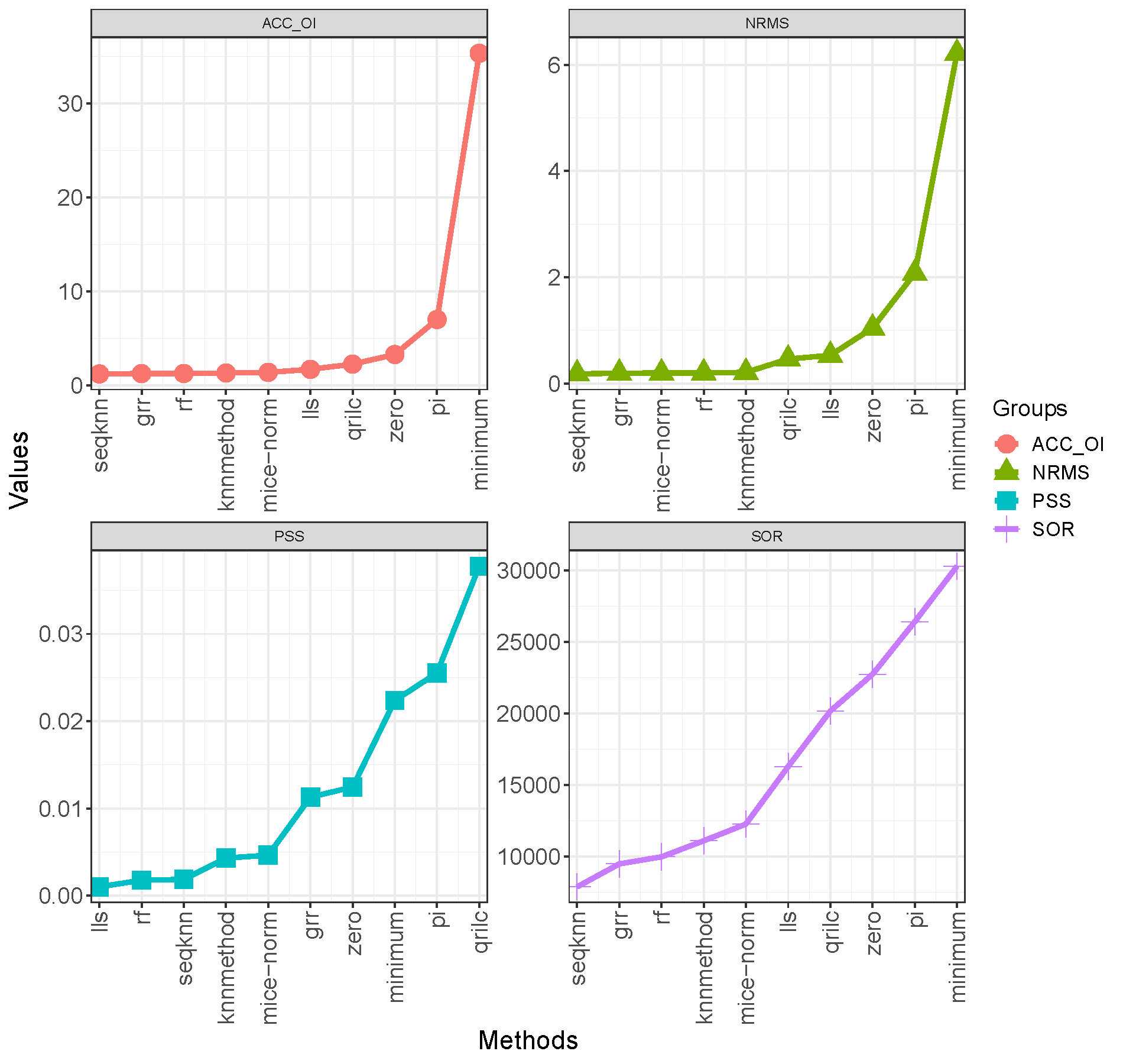


**Figure S2. Benchmarking of imputation methods for DIA dataset.** Ten imputation methods were assessed (zero, minimum, perseus imputation, K-nearest neighbor (knn), sequential knn (seqknn), quantile regression (qr), local least squares (lls), glmnet ridge regression (grr), multiple imputation bayesian linear regression (mice-norm), and random forest (rf)).


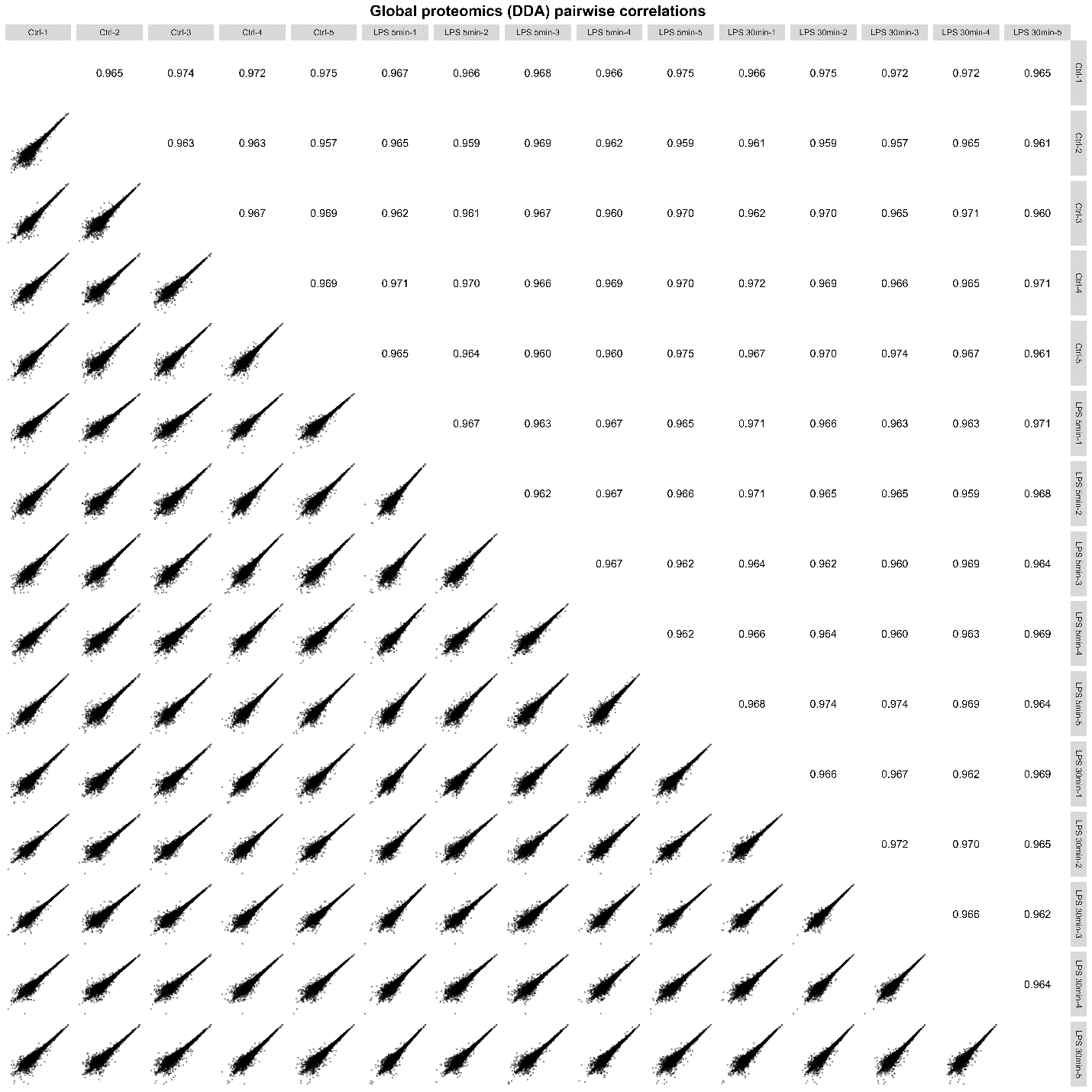


**Figure S3. Pairwise correlations between the samples in the global proteomics dataset using DDA.** Intensities were log_2_ transformed, and Pearson correlation coefficients are labeled in right panels. This plot was generated by R package (*ggplot2*).

**
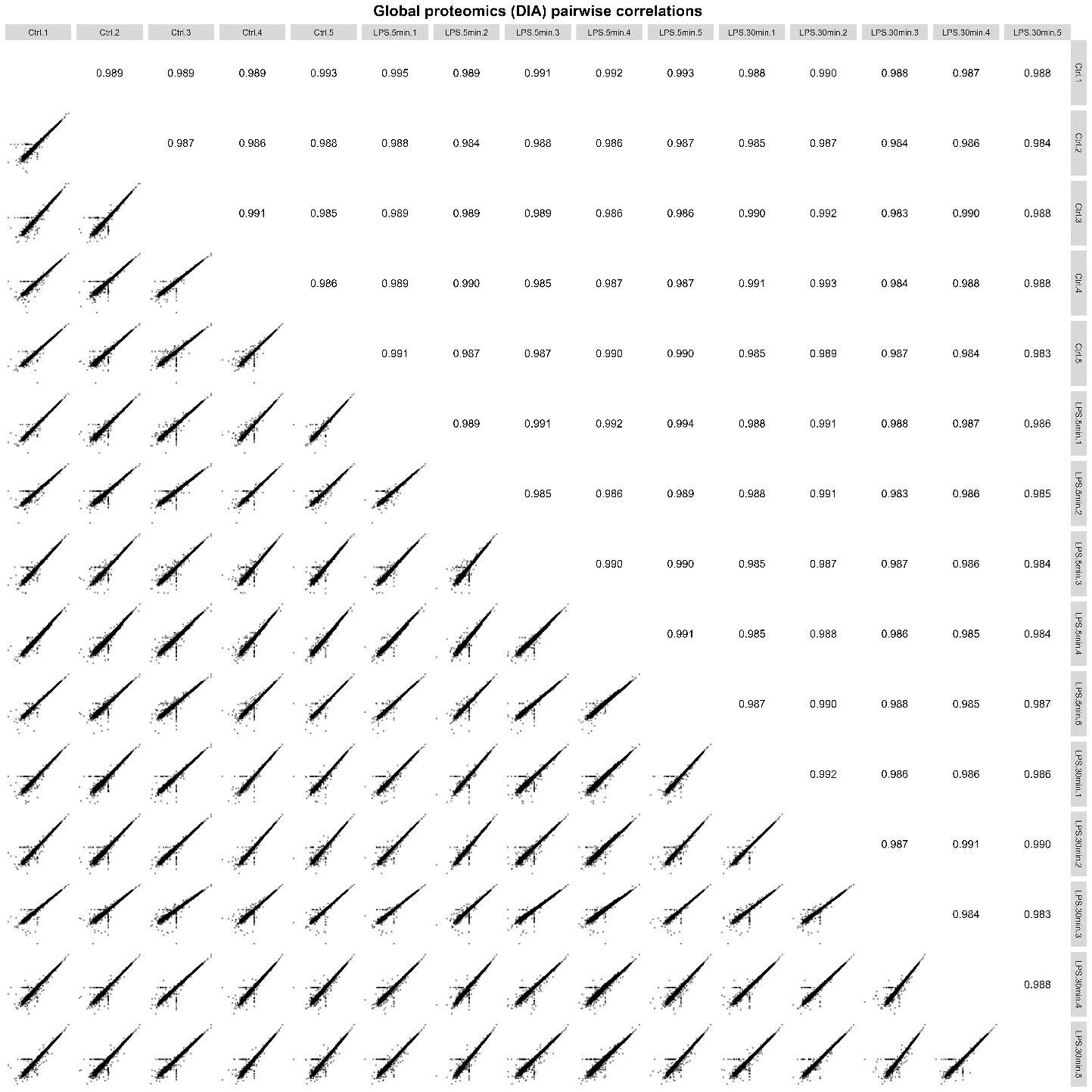
**

**Figure S4. Correlations between time-course LPS stimulation group in global proteomics dataset using DIA method.** Intensities were log_2_ transformed and Pearson correlation coefficients are labeled in right panels. This plot was generated by R package (*ggplot2*).


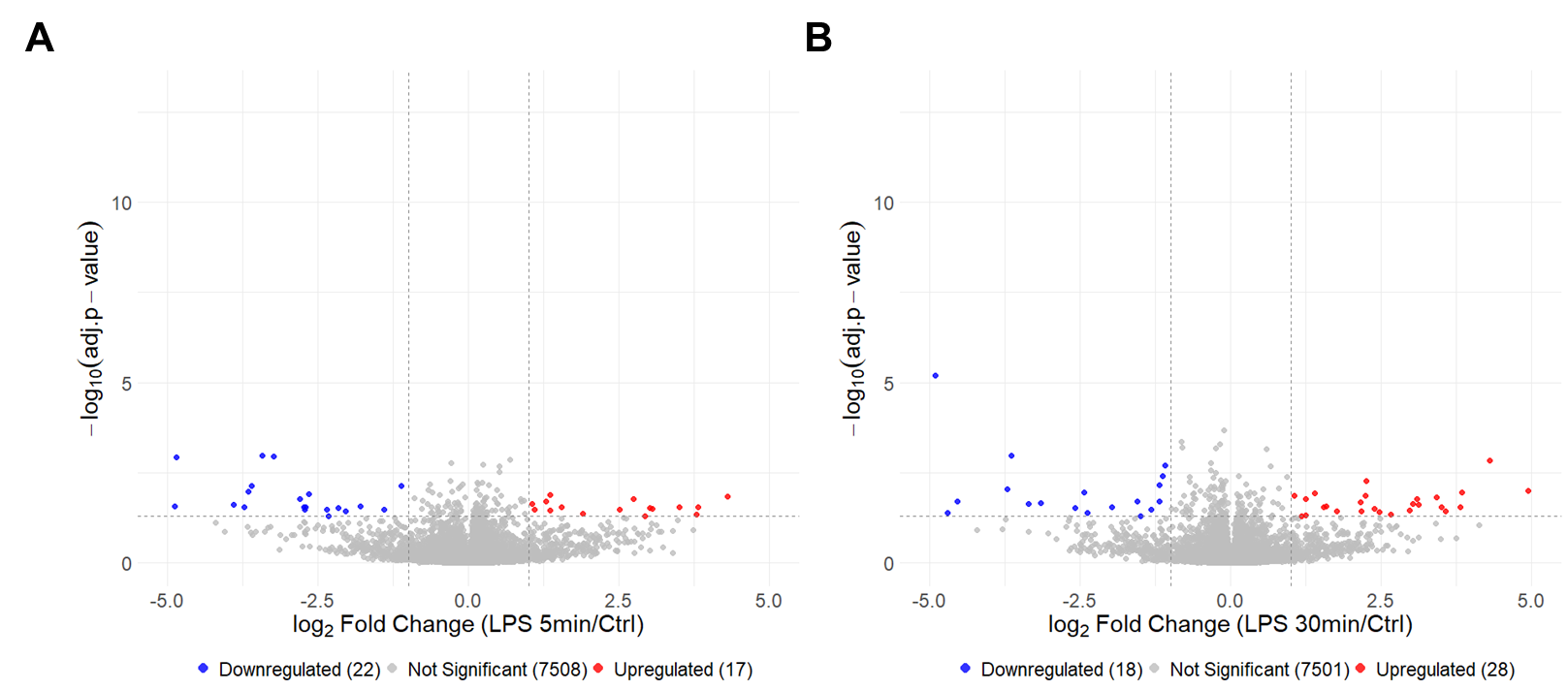


**Figure S5. Volcano plots of the global proteome DDA dataset.** **(A)** Volcano plot of the 5 min vs. Ctrl results. **(B)** Volcano plot of the 30 min vs. Ctrl results. The significance cut-off was set to +1 and -1 for the log_2_ abundance ratios, and adjusted p-value < 0.05. The blue dots indicate significantly downregulated proteins, and the red dots represent significantly upregulated proteins.


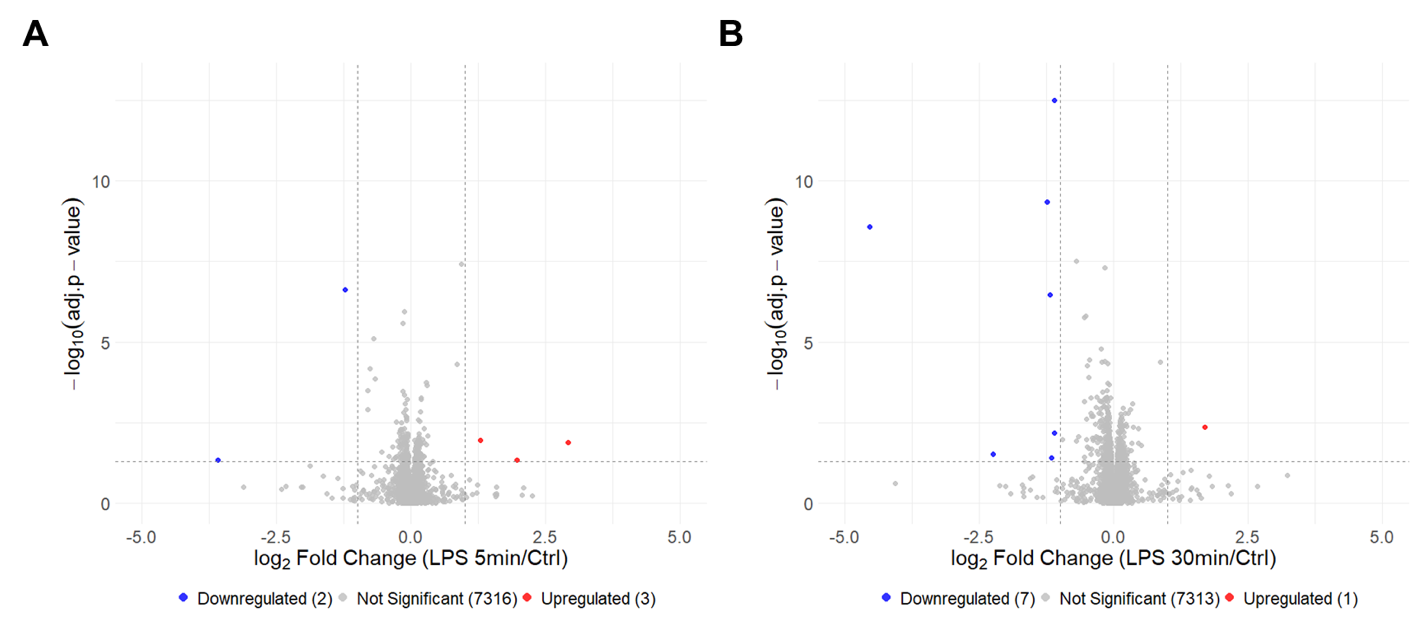


**Figure S6. Volcano plot of global proteome changes of DIA dataset in time-course LPS stimulated mouse macrophages.** **(A)** Volcano plot of LPS 5min/Ctrl with five biological replicates. **(B)** Volcano plot of LPS 30min/Ctrl with five biological replicates. Significance cut-off set to +1 and -1 for the log_2_ abundance ratio and adjusted p-value < 0.05. Blue dot indicates significantly downregulated proteins, and red dot represents significantly upregulated proteins.

**
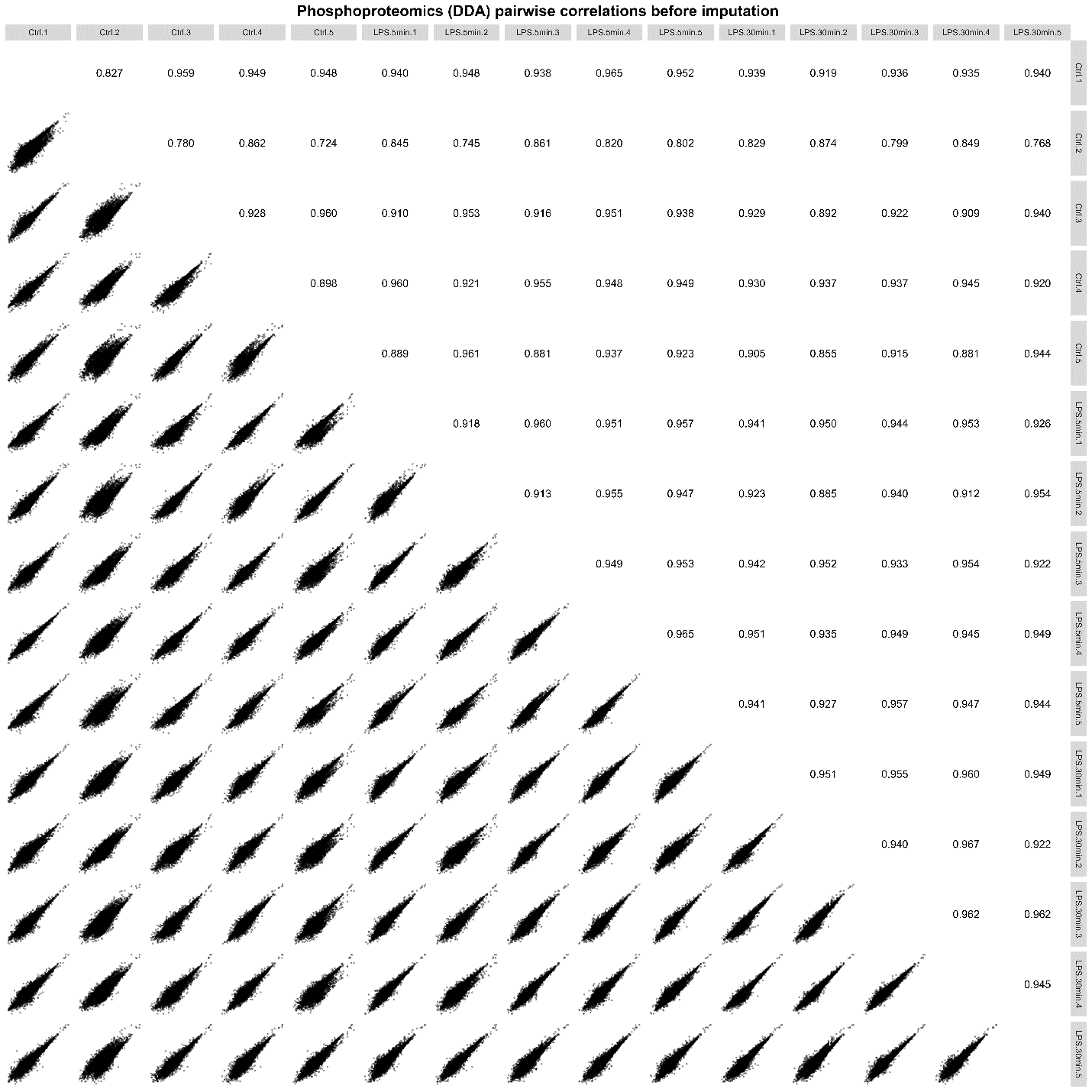
**

**Figure S7. Correlations between time-course LPS stimulation group in DDA phosphoproteomics dataset before imputation. Here, the primary matrix was used.** Intensities were log_2_ transformed and Pearson correlation coefficients are labeled in right panels. This plot was generated by R package (*ggplot2*).

**
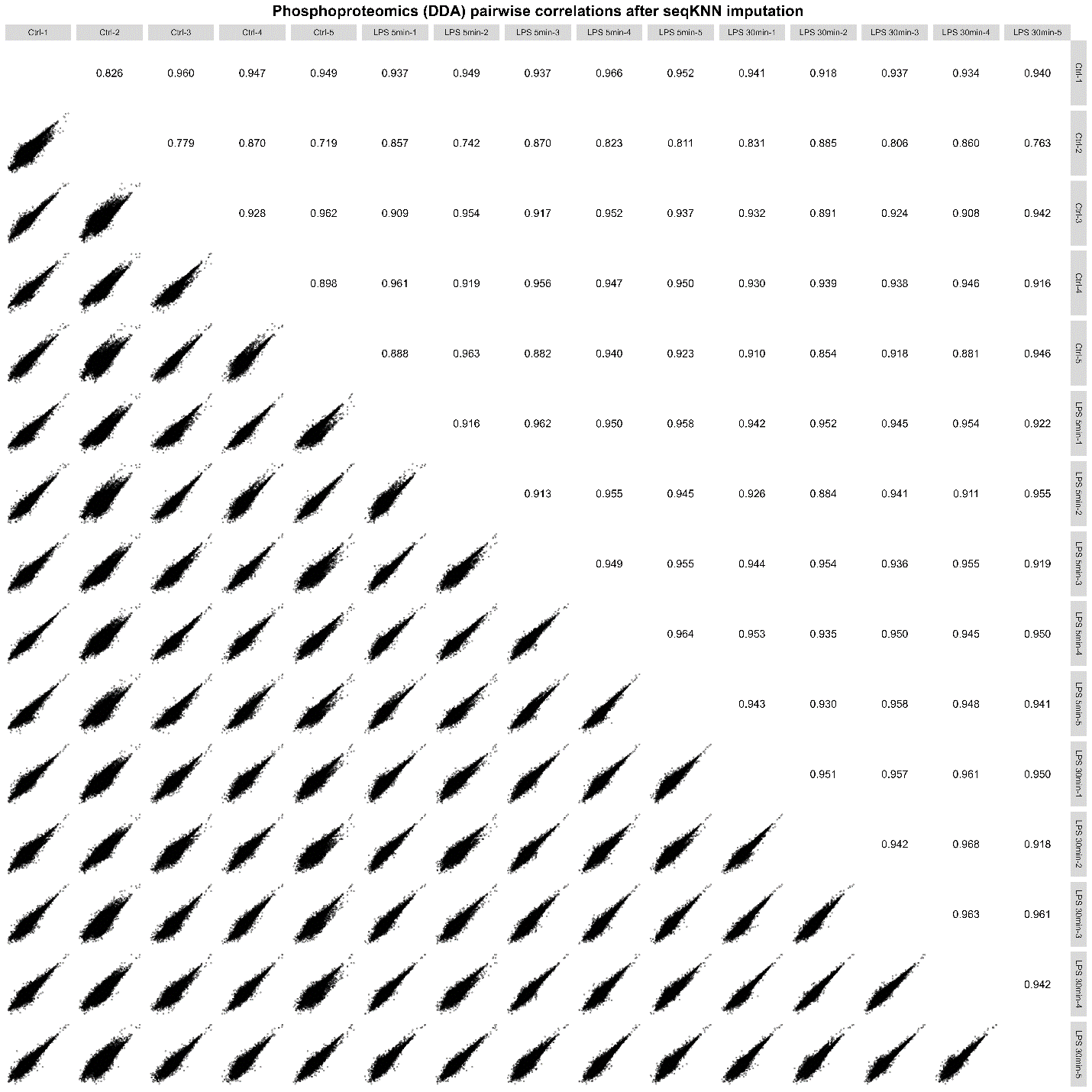
**

**Figure S8. Correlations between time-course LPS stimulation group in DDA phosphoproteomics dataset after seqKNN imputation. Here, the primary matrix was used.** Intensities were log_2_ transformed and Pearson correlation coefficients are labeled in right panels. This plot was generated by R package (*ggplot2*).


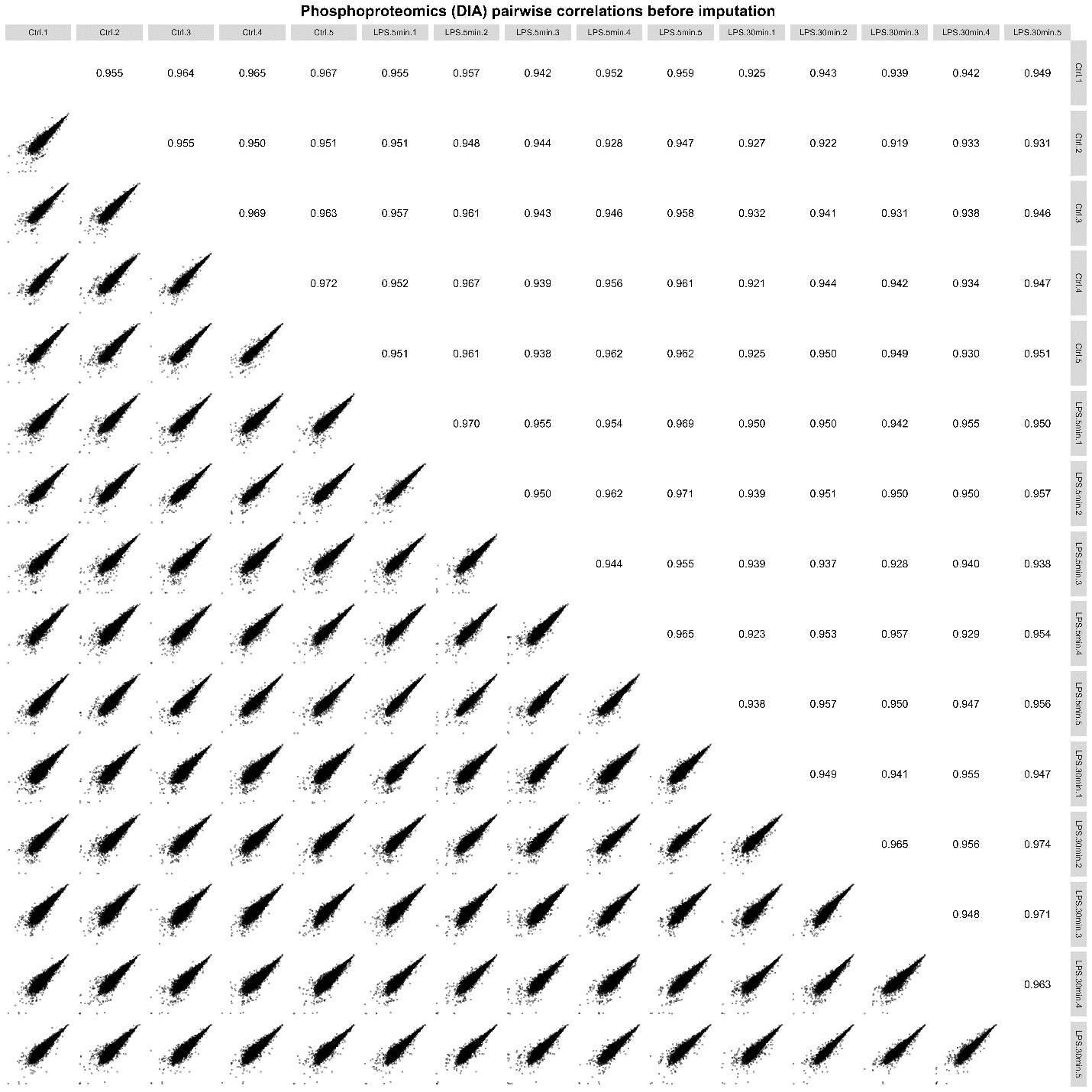


**Figure S9. Correlations between time-course LPS stimulation group in DIA phosphoproteomics dataset before imputation. Here, the primary matrix was used.** Intensities were log_2_ transformed and Pearson correlation coefficients are labeled in right panels. This plot was generated by R package (*ggplot2*).

**
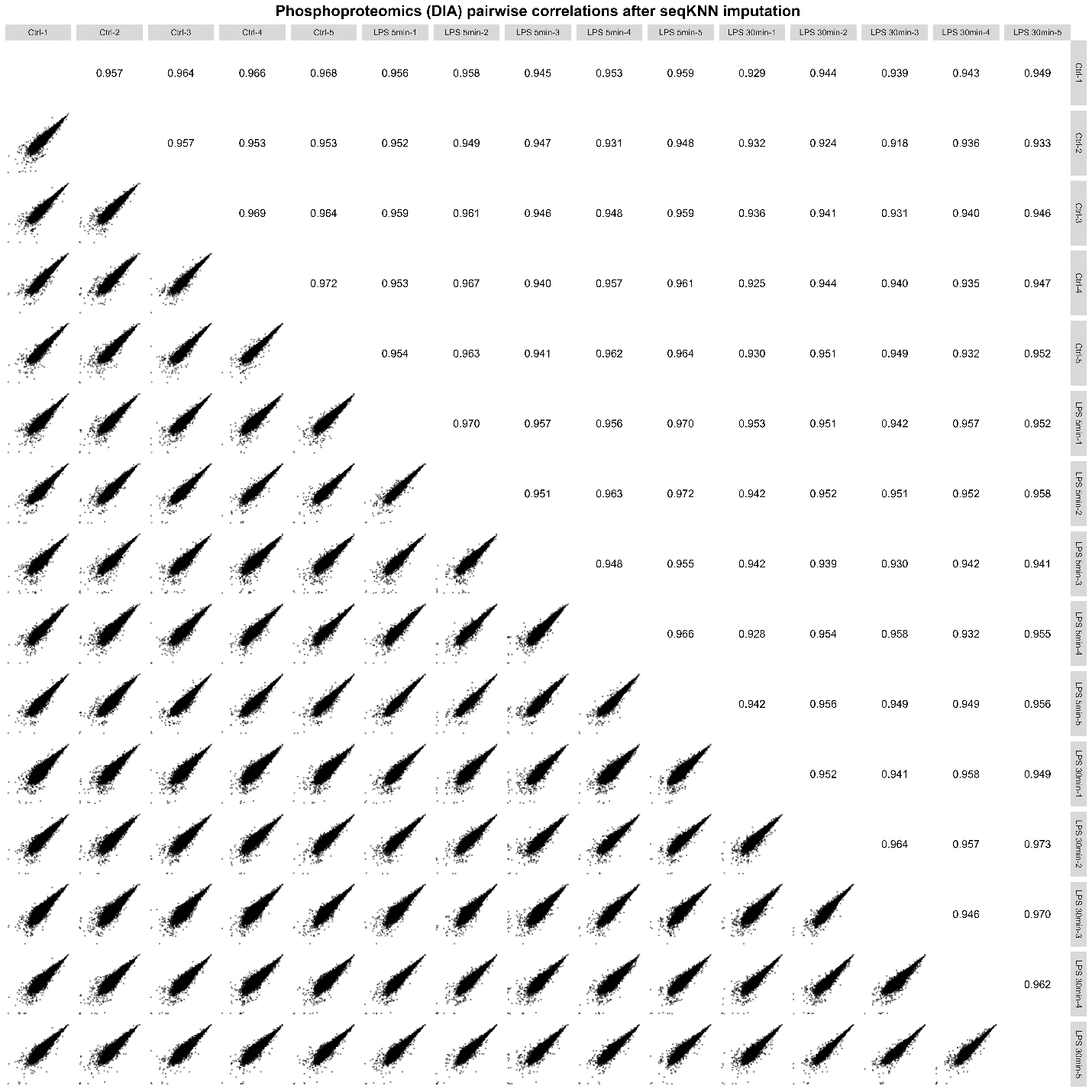
**

**Figure S10. Correlations between time-course LPS stimulation group in DIA phosphoproteomics dataset after seqKNN imputation. Here, the primary matrix was used.** Intensities were log_2_ transformed and Pearson correlation coefficients are labeled in right panels. This plot was generated by R package (*ggplot2*).


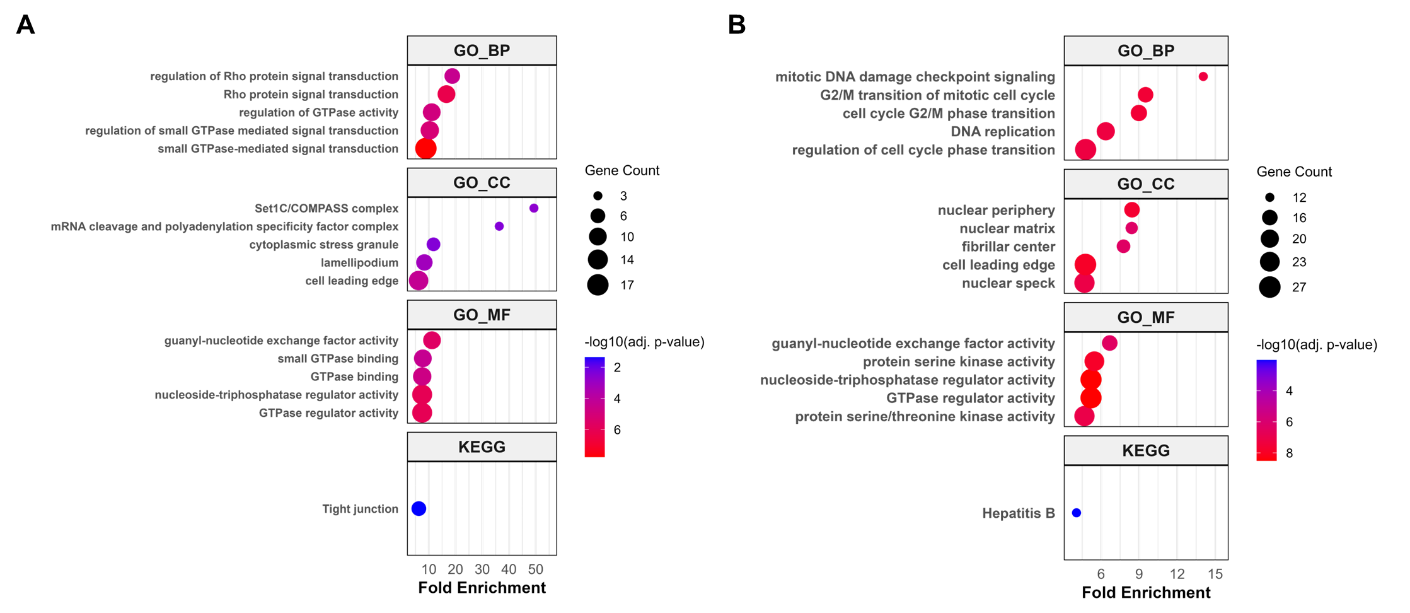


**Figure S11. GO and KEGG enrichment analyses results for significantly regulated phosphoproteins in 5 min LPS stimulation group.** Here, the primary data matrices were analyzed. (**A**) Dot plots of the Gene Ontology (GO) and (KEGG) enrichment results from significantly of the regulated phosphoproteins in the DDA dataset. (**B**) Dot plots of the GO and KEGG enrichment results from significantly regulated phosphoproteins in the DIA dataset. GO biological process (GO_BP), GO cellular components (GO_CC), GO molecular functions (GO_MF), and KEGG enrichment analyses were conducted using clusterProfiler.
